# Comprehensive gene expression analysis in gallbladder mucosal epithelial cells of dogs with gallbladder mucocele

**DOI:** 10.1111/jvim.17157

**Published:** 2024-11-12

**Authors:** Itsuma Nagao, Tomoki Motegi, Yuko Goto‐Koshino, Masaya Tsuboi, Naohiro Takahashi, James K. Chambers, Kazuyuki Uchida, Kenji Baba, Hirotaka Tomiyasu, Masaru Okuda

**Affiliations:** ^1^ Laboratory of Veterinary Internal Medicine, Department of Veterinary Medical Sciences, Graduate School of Agricultural and Sciences The University of Tokyo Tokyo Japan; ^2^ Department of Medicine, Division of Computational Biomedicine Boston University Chobanian & Avedisian School of Medicine Boston Massachusetts USA; ^3^ Laboratory of Veterinary Pathology, Graduate School of Agricultural and Life Sciences The University of Tokyo Tokyo Japan; ^4^ Laboratory of Veterinary Internal Medicine, Joint Faculty of Veterinary Medicine Yamaguchi University Yamaguchi Japan

**Keywords:** ANO1, gallbladder epithelial cells, HTR4, RNA‐seq

## Abstract

**Background:**

Gallbladder mucocele (GBM) is a common disease in the canine gallbladder. Although the pathogenesis of GBM remains unclear, we recently reported that the excessive accumulation of mucin in the gallbladder is not a result of overproduction by gallbladder epithelial cells (GBECs).

**Hypothesis/Objectives:**

Changes in the function of GBECs other than the production of mucin are associated with the pathogenesis of GBM. We performed an RNA sequencing (RNA‐seq) analysis to comprehensively search for abnormalities in gene expression profiles of GBECs in dogs with GBM.

**Animals:**

Fifteen dogs with GBM and 8 dogs euthanized for reasons other than gallbladder disease were included.

**Methods:**

The GBECs were isolated from gallbladder tissues to extract RNA. The RNA‐seq analysis was performed using the samples from 3 GBM cases and 3 dogs with normal gallbladders, and the gene expression profiles were compared between the 2 groups. Differences in mRNA expression levels of the extracted differentially expressed genes (DEGs) were validated by quantitative reverse transcription polymerase chain reaction (RT‐qPCR) using samples of 15 GBM cases and 8 dogs with normal gallbladders.

**Results:**

Comparison of gene expression profiles by RNA‐seq extracted 367 DEGs, including *ANO1*, a chloride channel associated with changes in mucin morphology, and *HTR4*, which regulates the function of chloride channels. The *ANO1* and *HTR4* genes were confirmed to be downregulated in the GBM group by RT‐qPCR.

**Conclusions and Clinical Importance:**

Our results suggest that GBM may be associated with decreased function of chloride channels expressed in GBECs.

**Abbreviations**
CF: cystic fibrosis
CFTR: cystic fibrosis transmembrane conductance regulator
DAVID: Database for Annotation, Visualization, and Integrated Discovery
DEGs: differentially expressed genes
FDR: false discovery rate
GBECs: gallbladder epithelial cells
GBM: gallbladder mucocele
GO: gene ontology
IP3: inositol 1,4,5‐triphosphate
KEGG: Kyoto Encyclopedia of Genes and Genomes
PLC: phospholipase C
RIN: RNA integrity number
RNA‐seq: RNA sequencing

## 1 INTRODUCTION

Gallbladder mucocele (GBM) is a common gallbladder disease in dogs.^1^ It is characterized by excessive accumulation of mucus, mainly mucin, in the gallbladder and can cause bile duct obstruction and rupture of the gallbladder.^2^, ^3^ The only treatment available is surgery, which has a high perioperative mortality rate of approximately 20%.^4^, ^5^, ^6^ Several epidemiologic studies have reported that several breeds such as Shetland Sheepdogs, American Cocker Spaniels, Chihuahuas, Pomeranians, Miniature Schnauzers, and Border Terriers^7^, ^8^, ^9^, ^10^ were predisposed to develop GBM. Also, endocrinopathies such as hyperadrenocorticism, hypothyroidism, and hyperlipidemia^9^, ^11^ are reported to be risk factors for the development of GBM, but the pathogenesis of GBM has not yet been elucidated.

In the gallbladder, mucins are secreted by the gallbladder epithelial cells (GBECs) to protect the mucosa from the surfactant effects of bile.^12^, ^13^ It has been reported that the gallbladder expresses MUC1, MUC2, MUC3, MUC4, MUC5AC, MUC5B, and MUC6 in dogs, mice, and humans.^14^, ^15^, ^16^, ^17^, ^18^ Furthermore, a study using mass spectrometry confirmed that MUC5AC and MUC5B are the most abundant mucin subtypes in the mucus of GBM cases and reported that MUC5AC concentrations increased compared with healthy dogs.^13^ However, we previously reported that the expression levels of MUC5AC and MUC5B were not increased in the GBECs at both the gene and protein levels,^18^ indicating that excess mucus accumulation might be a result of impaired mucus excretion rather than excessive production. Hence, it is necessary to investigate factors that cause abnormalities in mucus excretion.

Mucin, a large molecule consisting of a heavily glycosylated core protein, is stored within the secretory granules in a highly folded compact form before secretion (ie, packed mucins). As they are secreted into the gallbladder lumen, the packed mucins are gradually unpacked into a linear form and excreted from the gallbladder. In a previous study analyzing the molecular structure of mucin in the gallbladder of dogs, mucin from healthy dogs had a linear molecular morphology, whereas mucin from GBM had a compact morphology with many intramolecular cross‐links,^13^ suggesting abnormalities in the unpacking process of mucins in GBM cases. Combined with the fact that GBECs not only secrete mucin but also are involved in the unpacking process of mucin by regulating the ionic composition of bile and water content,^17^ their functions may be involved in the changes in mucin morphology observed in the mucus of the GBM. We aimed to identify candidate proteins responsible for the accumulation of mucus in GBM by comprehensively analyzing gene expression profiles in GBECs, which produce mucin and have a role in regulating mucin properties.

## 2 MATERIALS AND METHODS

### 2.1 Animals

Dogs that underwent cholecystectomy at the Veterinary Medical Center of the University of Tokyo and were histopathologically diagnosed with GBM at the Laboratory of Veterinary Pathology at the University of Tokyo were included in the study. The diagnosis of GBM was made based on gross examination and light microscopy of gallbladder, as in previous studies,^10^, ^13^, ^19^ which identified abundant mucus accumulation in the lumen, hyperplasia of the mucosal epithelial cells, and their elongation into a columnar shape in all cases included in the present study. All owners provided written informed consent for the use of gallbladder tissues. Normal canine gallbladders were obtained from research animals maintained at Yamaguchi University that were euthanized for other experimental purposes approved by the Yamaguchi University Ethics Committee (Approval No. 209). The age of these healthy dogs ranged from 6 to 8 years. The experimental procedures and animal care were conducted in conformity with the Yamaguchi University Animal Care and Use guidelines.

### 2.2 Collection of GBECs

Isolation of GBECs was performed as previously reported.^18^ Gallbladder tissues were obtained during surgical cholecystectomy from GBM cases or collected from research dogs immediately after euthanasia. The gallbladder was cut open and washed with ice‐cold phosphate‐buffered saline (PBS). Any remaining mucus gel was removed using forceps. Then, gallbladder tissues were digested using 0.25% w/v trypsin (Fujifilm Wako, Osaka, Japan) or 2000 U/mL of dispase (Goudou Shusei, Tokyo, Japan) at 37°C for 45 minutes to collect GBECs. After inactivation of trypsin by adding 10% fetal bovine serum, remaining debris was removed by passing through a 100‐μm cell strainer, followed by centrifugation at 1500 rpm for 5 minutes. After another wash with PBS, the pelleted cells were stored at −80°C until utilized. The viability of GBECs was confirmed to be >95% using the trypan blue exclusion test.

### 2.3 RNA sequencing analysis

Total RNA was extracted from GBECs using RNeasy mini kit (Qiagen, Hilden, Germany) following the manufacturer's protocol. The RNA integrity number (RIN) was assessed using an Agilent 2100 Bioanalyzer (Agilent Technologies, Santa Clara, California, USA), and RNA with an RIN >7 was used for RNA sequencing (RNA‐seq). Sequencing libraries were prepared with the total RNA using NEBNext UltraTM ll Directional RNA Library Prep Kit (NEW ENGLAND Biolabs, Ipswich, Massachusetts, USA). RNA‐seq (150 bp paired‐end) was conducted using a NovaSeq 6000 (Illumina, San Diego, California, USA).

Quality controls and adaptor trimmings of the obtained fastq files were conducted using Fastp. Trimmed fastq data were mapped to canine genomes (CanFam3.1) by STAR 2.7.3a,^20^ and transcript abundance was estimated using RSEM v.1.3.3^21^ with gene transfer file for Ensembl (CanFam3.1.98, https://www.ensembl.org). These gene count data were used to normalize and extract differential gene expressions with an R package (DEseq2 V.1.32.0). Differentially expressed genes (DEGs) were identified by comparison between the 2 groups, normal and GBM, at a false discovery rate (FDR) < 0.2. To understand the underlying biological mechanisms and pathways associated with the identified DEGs, we performed gene ontology (GO) enrichment analysis and Kyoto Encyclopedia of Genes and Genomes (KEGG) pathway analysis using all DEGs, which were extracted from the comparison between the 2 groups by the Database for Annotation, Visualization, and Integrated Discovery (DAVID). The analysis using DAVID was conducted with the background gene list of standard option. To correct for multiple comparisons, the *P*‐values were adjusted by employing the Benjamini‐Hochberg procedure with a threshold of FDR < 0.05.

The datasets used and analyzed are available at the DDBJ Sequenced Read Archive repository with accession number PRJDB17836.

### 2.4 Quantitative reverse transcription polymerase chain reaction

Extracted total RNA was reverse transcribed using ReverTra Ace (TOYOBO, Osaka, Japan). The quantitative reverse transcription polymerase chain reaction (RT‐qPCR) was performed using StepOnePlus (Applied Biosystems, Waltham, Massachusetts, USA) with PowerUP SYBR Green Master Mix (Applied Biosystems). Relative mRNA amounts for each gene were calculated by standard curves and normalization was conducted by dividing them by the relative mRNA amount of *GAPDH* as the internal control. Each PCR reaction was performed in duplicate. The primers used are listed in Table S1.

### 2.5 Statistical analysis

Statistical analysis was performed using R v4.1.0 (R core team, Vienna, Austria). The Wilcoxon rank‐sum test with the exact test option was used for the comparison of relative amounts of mRNAs between the 2 groups. A *P*‐value of < .05 was considered significant.

## 3 RESULTS

### 3.1 Animals

Fifteen dogs with GBM and 8 normal dogs were included (Table S2). All of the cases included were diagnosed as GBM by pathological examination, in which 14 were also clinically diagnosed as GBM by ultrasound examination (Figure S1), but 1 case was clinically diagnosed as gallbladder sludge before histopathological examination. The GBM cases included 5 intact males, 1 intact female, 4 neutered males, and 5 spayed females. There were 5 Toy Poodles, 2 Chihuahuas, 2 Shetland Sheepdogs, 2 Papillons, 1 Miniature Schnauzer, 1 Beagle, 1 Maltese, and 1 Parson Russell terrier. In all cases, there was no rupture of the gallbladder, and no necrosis of the gallbladder wall was observed in histopathological examination. Normal gallbladders were collected from 8 beagles, which included 6 intact females and 2 intact males.

### 3.2 Extractions of DEGs in GBM and enrichment analysis

**FIGURE 1:**
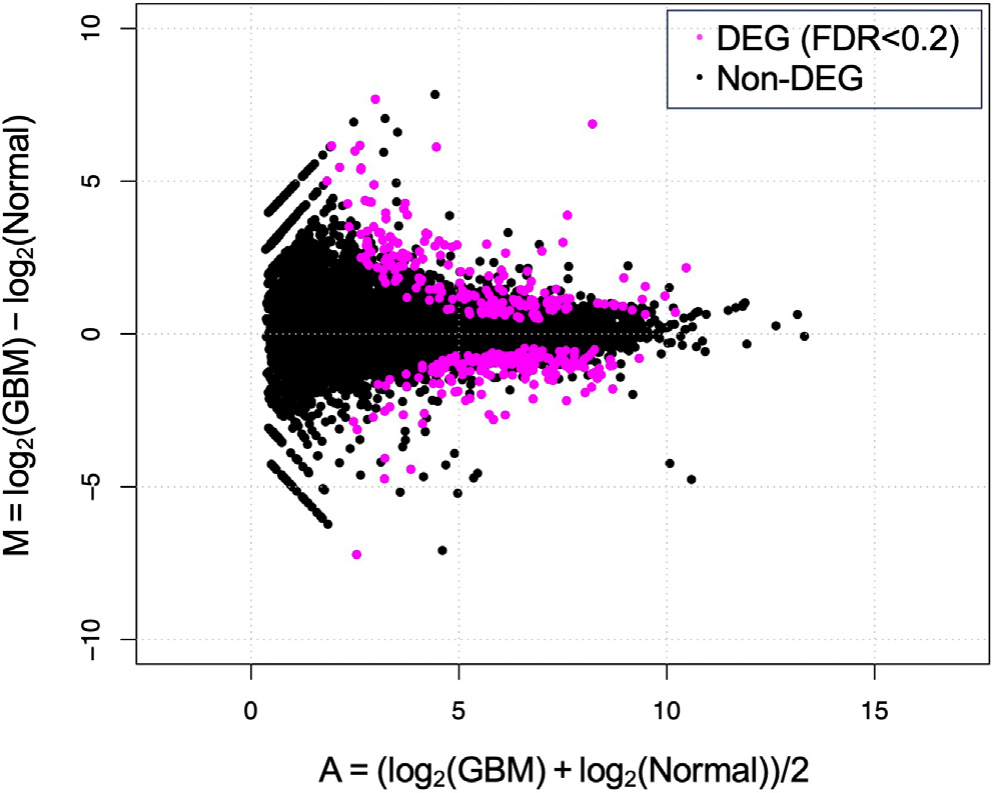
M‐A plot of differentially expressed genes (DEGs). Differentially expressed genes extracted from the comparison of 2 groups, dogs with gallbladder mucocele (GBM) and normal gallbladder, are plotted by M‐A plot. DEGs with false discovery rate (FDR) <0.2 are plotted in magenta.

The RNA‐seq analysis generated at least 27.93 million total raw reads for each paired end, with a mapping rate of at least 95.17% (Table S3). In a comparison between the 2 groups, normal and GBM, 367 DEGs were extracted at FDR < 0.2, of which 192 genes were upregulated and 175 genes were downregulated in the GBM group (Figure 1 and Table S4). Based on q‐values, the top 5 upregulated genes were *RIPPLY1*, *CCKAR*, *BICC1*, *PLK2*, and *MNDA*, whereas the top 5 downregulated genes were *CLEC3A*, *MT1E*, *ZDHHC7*, *TSPAN5*, and *HIF3A*. The expression levels of mucins were not different between the 2 groups except for *MUC1*, which was consistent with our previous study.^18^

The GO enrichment analysis using all DEGs was performed to investigate the associated biological functions according to 3 categories: biological process, cellular component, and molecular function (Table 1). Two and 8 significant terms were extracted for the biological process and cellular component, respectively, whereas no significant term was extracted for the molecular function. “Cholesterol biosynthetic process” and “Sterol biosynthetic process” were enriched in the biological process, but terms related to mucus or mucin were not enriched. In the KEGG pathway analysis, only the pathway “Steroid biosynthesis” was extracted (Gene count, 7; FDR = 0.00018).

**TABLE 1:** Results of gene ontology analysis using differentially expressed genes.

Category	Term	Count	FDR
Biological process	Cholesterol biosynthetic process	10	6.16E−06
Biological process	Sterol biosynthetic process	7	2.20E−04
Cellular component	Apical plasma membrane	23	5.54E−05
Cellular component	Endoplasmic reticulum	40	2.40E−04
Cellular component	Endoplasmic reticulum membrane	35	6.40E−03
Cellular component	Cytoplasm	116	6.49E−03
Cellular component	Cytosol	113	6.49E−03
Cellular component	Extracellular exosome	53	2.35E−02
Cellular component	Basolateral plasma membrane	13	2.35E−02
Cellular component	Integral component of plasma membrane	38	2.98E−02

Abbreviation: FDR, false discovery rate.

### 3.3 Validations of expression levels of DEGs using RT‐qPCR

**FIGURE 2:**
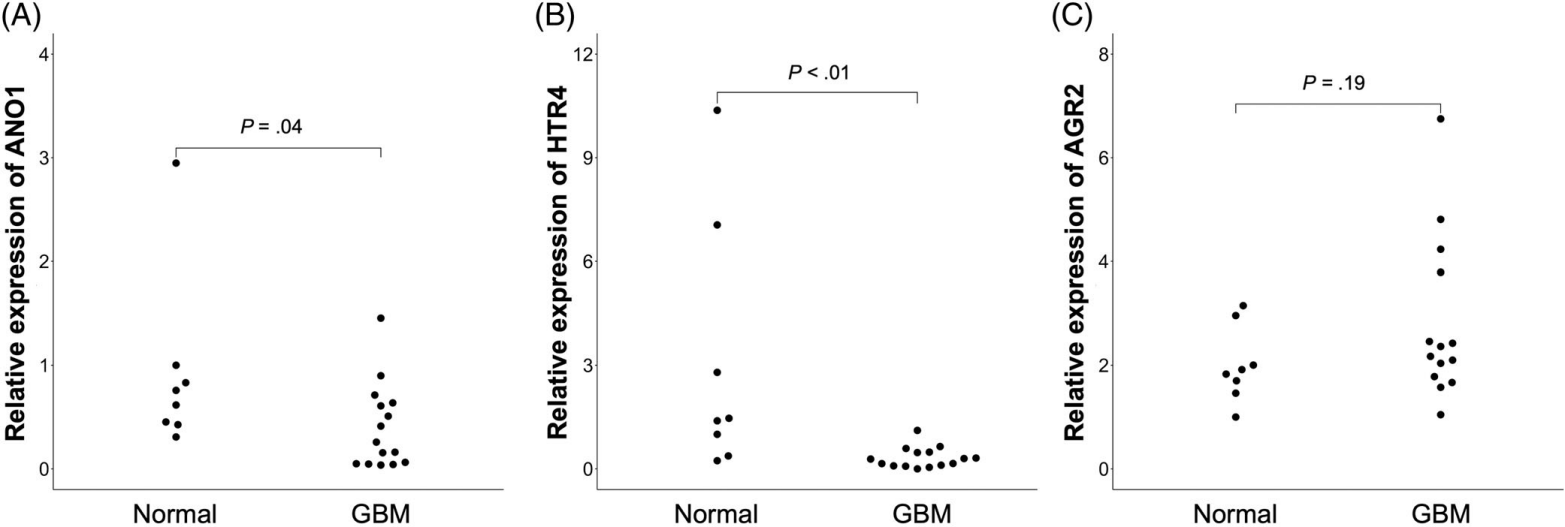
Comparison of the gene expression levels of differentially expressed genes using quantitative reverse transcription polymerase chain reaction (RT‐qPCR). Among differentially expressed genes extracted by RNA‐seq, gene expression levels of *ANO1* (A), *HTR4* (B), and *AGR2* (C), which were included in the term “mucus secretion” in gene ontology, were quantified by RT‐qPCR using 8 normal dogs and 15 gallbladder mucocele (GBM) cases.

Because no pathways involving mucus or mucin were extracted in the enrichment analysis, we focused on DEGs included in “mucus secretion” in the biological process of the GO term, although it was not included in the terms significantly enriched with DEGs. Among the DEGs, 3 genes were included in “mucus secretion”: *AGR2*, *ANO1*, and *HTR4*, and their mRNA expression levels were compared using RT‐qPCR between the 2 groups: normal and GBM groups. Although no difference was found in *AGR2* mRNA expression between the 2 groups (*P* = .19), *ANO1* and *HTR4* mRNA expression was significantly decreased in the GBM group (*P* = .04, and *P* < .01, respectively; Figure 2), consistent with the RNA‐seq results.

## 4 DISCUSSION

Gallbladder mucocele is characterized by excessive mucus accumulation in the gallbladder, substantially impacting its function. To identify the cause of excessive mucin accumulation in GBM cases, we performed a comprehensive analysis of gene expression profiles in GBECs of GBM cases and healthy dogs, that secrete mucin and are responsible for promoting its morphological changes. In a comparison between GBM cases and normal dogs, 367 genes were extracted as DEGs, which included genes involved in mucus secretion, such as *ANO1*, *HTR4*, and *AGR2*. In the validation using RT‐qPCR, expression levels of *ANO1* and *HTR4* were confirmed to be downregulated in dogs with GBM compared with normal dogs.

Anoctamin 1, encoded by *ANO1*, is a Ca^2+^‐activated chloride channel, which is an ion channel that secretes anions such as chloride and bicarbonate from the intracellular to the extracellular space upon an increase of intracellular calcium ion concentration.^22^ Because Anoctamin 1 is responsible for anion secretion in epithelial cells,^23^, ^24^ decreased expression of *ANO1* may lead to decreased anion secretion from GBECs. Mucin is packed in a folded structure in intracellular secretory granules with the calcium ions cross‐linking between the sugar chains of mucin.^17^ As mucin is secreted, calcium ions are replaced by sodium ions and chelated by bicarbonate ions in bile, transforming the packed mucins into linear‐formed fluid mucins with cleaved cross‐links.^25^ It has been reported that dysfunction of the cystic fibrosis transmembrane conductance regulator (CFTR), another major chloride channel, causes insufficient anion secretion and defects in the unpacking process of mucin, resulting in mucus accumulation in the gastrointestinal tract and airways in humans with cystic fibrosis (CF).^26^ Dysfunction of CFTR or decreased expression in the gallbladder has not been reported in GBM before, and the expression level of *CFTR* was not different between GBM and normal canine gallbladder in our study. However, our results suggest that similar changes as observed in CF patients also may have occurred in the gallbladder of GBM cases because of decreased expression of *ANO1*. Furthermore, a report analyzing the mucus secreted from the gallbladder showed that linear‐formed fluid mucin constitutes the majority in healthy dogs, whereas granular mucin with a compact morphology accounts for the majority of the mucin in GBM patients,^13^ which indicates defects in the unpacking process of mucin. Hence, a decrease in *ANO1* expression may cause a reduction in chloride ion concentrations in the bile, leading to abnormalities in the process of mucin unpacking.

5‐hydroxytryptamine receptor 4 (HTR4), which is encoded by *HTR4*, is the G protein‐coupled receptor (GPCR) that stimulates cAMP production in response to serotonin binding.^27^ Because our study showed a decrease in *HTR4* expression, a concomitant decrease in intracellular cAMP concentration can be inferred, which is consistent with the significantly decreased cAMP concentration in bile of dogs with GBM compared with healthy dogs in a study that analyzed the metabolites in bile of dogs with GBM.^28^ It has been reported that cAMP produced by HTR4 activates CFTR, a cAMP‐activated chloride channel, promoting anion secretion.^29^, ^30^ In addition, GPCR induces the release of calcium from the endoplasmic reticulum through phospholipase C (PLC)/inositol 1,4,5‐triphosphate (IP3) signaling, which increases intracellular calcium concentration and evokes activation of anoctamin 1.^22^, ^31^ Therefore, decreased expression of HTR4 may suppress cAMP‐mediated activation of CFTR and PLC/IP3 pathway‐mediated activation of anoctamin 1, resulting in decreased anion secretion into the bile.

Several studies have pointed out the similarity between GBM and CF, where decreased anion secretion caused by abnormal CFTR function results in excessive mucus accumulation. It has been reported that *CFTR*‐knock‐out models in pigs and ferrets, similar to GBM, show mucus accumulation in the gallbladder and characteristic papillary elongation of the mucosal epithelium on pathological examination.^32^, ^33^ However, because CF is a disease caused by congenital genetic mutations, it often develops at a young age, but GBM develops at a middle to older age.^8^, ^34^ Furthermore, it is known that people with CF do not show enlargement of the gallbladder associated with mucus retention as in GBM. Rather, microgallbladders with an accumulation of highly gelled mucus often are observed.^35^, ^36^ Our results suggest that GBM could be caused by abnormal ion secretory function, which is similar to CF in humans, but it is necessary to investigate GBM‐specific mechanisms considering these differences between CF and GBM.

In our study, the number of DEGs extracted by RNA‐seq analysis was 367 at FDR <0.2. This result indicated that the gene expression profiles did not change markedly despite the severe pathological changes in the gallbladder tissues when compared with the results of other studies that performed RNA‐seq in dogs with various diseases and in healthy dogs.^37^, ^38^, ^39^ Possible causes of this result include a large variation in gene expression profiles of the GBECs among the cases, which masks GBM‐specific changes in gene expression. Although GBM cases included in the present RNA‐seq analysis did not have any concomitant diseases, it is possible that diet and medications, which are reported to affect the composition of bile,^40^, ^41^, ^42^, ^43^ might have changed gene expression in GBECs through alterations in the composition of the bile. It also might be possible that alterations related to GBM pathogenesis cause little change in the gene expression profiles of GBECs. An RNA‐seq analysis using GBECs of *CFTR* knockout pigs has reported only 163 DEGs.^44^ In this previous study, pathways involving *CFTR* gene were not extracted in the gene enrichment analysis, suggesting little impact of the knocked‐out of *CFTR* gene expression on comprehensive gene expression profiles although alteration of ion exchange procedures occurred.^44^ Because *ANO1*, detected as a DEG in our study, is also a gene involved in anion exchange as is *CFTR*, changes in *ANO1* expression may have as little effect on the expression profiles of other genes as in *CFTR* knock‐out pigs.

In our study, RNA‐seq was performed on 3 dogs with GBM and 3 healthy dogs, and qPCR validation also was conducted on 15 dogs with GBM and 8 healthy dogs. All healthy dogs were Beagles, whereas the dogs with GBM consisted of 8 different breeds. Because the predicted pathogenesis of the disease was based on only 8 breeds in our study, a limitation of the study is that breed‐specific factors might have been masked because of the different genetic backgrounds of the breeds. Therefore, a larger number of GBM cases should be studied and breed‐specific analysis should be conducted in the future.

In conclusion, we found that expression of genes involved in anion channel function, such as *ANO1* and *HTR4*, were downregulated in GBM cases, suggesting that decreased anion secretion may lead to an abnormal mucin unpacking process and mucus accumulation. Ion channel function should be further evaluated using a cell culture system to confirm that anion secretion is decreased in GBM cases.

## CONFLICT OF INTEREST DECLARATION

Authors declare no conflict of interest.

## OFF‐LABEL ANTIMICROBIAL DECLARATION

Authors declare no off‐label use of antimicrobials.

## INSTITUTIONAL ANIMAL CARE AND USE COMMITTEE (IACUC) OR OTHER APPROVAL DECLARATION

Approved by the IACUC of Yamaguchi University (Approval Number 209).

## HUMAN ETHICS APPROVAL DECLARATION

Authors declare human ethics approval was not needed for this study.

## Supporting information


**Figure S1.** Representative images of ultrasound and histopathological examinations of GBM cases included in the present study. Results of ultrasound and histopathological examinations of the gallbladders of GBM2, GBM5, and GBM14.

**Table S1.** Primer pairs used in RT‐qPCR for the 4 genes.

**Table S2.** Signalment of the cases with gallbladder mucocele included in the present study. Abbreviation: GBM, gallbladder mucocele.

**Table S3.** Total read counts and Mapping rate obtained by RNA sequencing.
Abbreviations: GBM, gallbladder mucocele.

**Table S4.** Differentially expressed genes extracted by comparison between gallbladder mucocele cases and healthy dogs. Abbreviation: FDR, false discovery rate.
