# Supplementary figures and images for: Comprehensive gene expression analysis in gallbladder mucosal epithelial cells of dogs with gallbladder mucocele
Source: J Vet Intern Med. 2024 Nov 12;38(6):3031–7. doi: 10.1111/jvim.17157 (PMC11586547; doi:10.1111/jvim.17157)

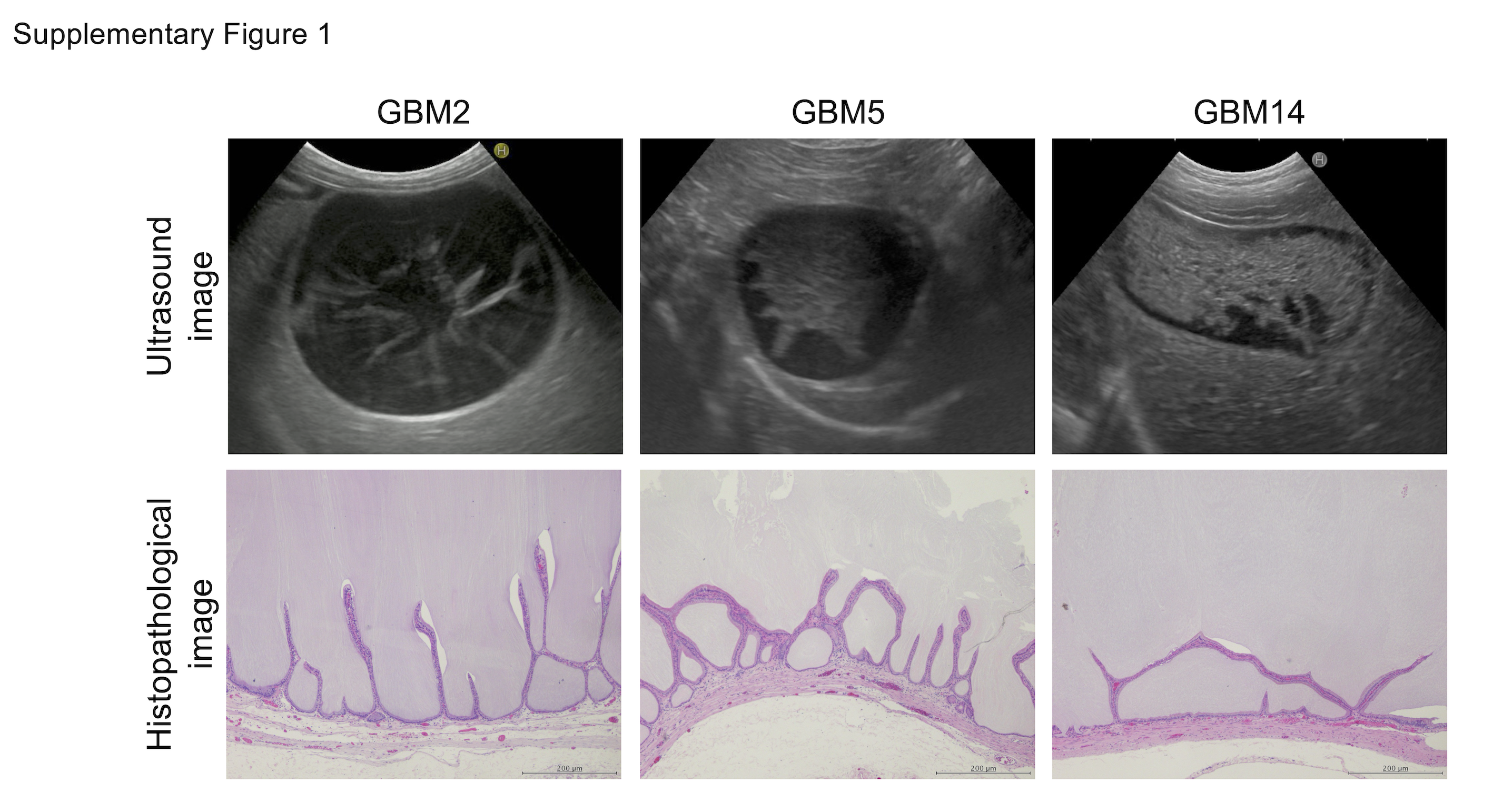

Supplement: Supplementary file 1 — Figure S1. Representative images of ultrasound and histopathological examinations of GBM cases included in the present study. Results of ultrasound and histopathological examinations of the gallbladders of GBM2, GBM5, and GBM14. [file JVIM-38-3031-s003.tiff]
